# Ultra‑Broadband and Ultra-High Electromagnetic Interference Shielding Performance of Aligned and Compact MXene Films

**DOI:** 10.1007/s40820-025-01750-z

**Published:** 2025-04-27

**Authors:** Weiqiang Huang, Xuebin Liu, Yunfan Wang, Jiyong Feng, Junhua Huang, Zhenxi Dai, Shaodian Yang, Songfeng Pei, Jing Zhong, Xuchun Gui

**Affiliations:** 1 State Key Laboratory of Optoelectronic Materials and Technologies, School of Electronics and Information Technology, Sun Yat-Sen University, Guangzhou, 510275 People’s Republic of China; 2 National Key Laboratory of Materials for Integrated Circuits, Shenzhen Institute of Advanced Electronic Materials, Shenzhen Institute of Advanced Technology, Chinese Academy of Sciences, Shenzhen, 518055 People’s Republic of China; 3 Shenyang National Laboratory for Materials Science, Institute of Metal Research, Chinese Academy of Sciences, 72 Wenhua Road, Shenyang, 110016 People’s Republic of China; 4 School of Materials Science and Engineering, University of Science and Technology of China, 72 Wenhua Road, Shenyang, 110016 People’s Republic of China; 5 Key Lab of Structure Dynamic Behavior and Control (Harbin Institute of Technology), Ministry of Education, Harbin, 150090 People’s Republic of China

**Keywords:** MXene Film, Electromagnetic interference shielding, Infrared stealth, Electrical heater

## Abstract

With the rapid development of electronic detective techniques, there is an urgent need for broadband (from microwave to infrared) stealth of aerospace equipment. However, achieving effective broadband stealth primarily relies on the composite of multi-layer coatings of different materials, while realizing broadband stealth with a single material remains a significant challenge. Herein, we reported a highly compact MXene film with aligned nanosheets through a continuous centrifugal spraying strategy. The film exhibits an exceptional electromagnetic interference shielding effectiveness of 45 dB in gigahertz band (8.2–40 GHz) and 59 dB in terahertz band (0.2–1.6 THz) at a thickness of 2.25 μm, owing to the high conductivity (1.03 × 10^6^ S m^−1^). Moreover, exceptionally high specific shielding effectiveness of 1.545 × 10^6^ dB cm^2^ g⁻^1^ has been demonstrated by the film, which is the highest value reported for shielding films. Additionally, the film exhibits an ultra-low infrared emissivity of 0.1 in the wide-range infrared band (2.5–16.0 μm), indicating its excellent infrared stealth performance for day-/nighttime outdoor environments. Moreover, the film demonstrates efficient electrothermal performance, including a high saturated temperature (over 120 °C at 1.0 V), a high heating rate (4.4 °C s^−1^ at 1.0 V), and a stable and uniform heating distribution. Therefore, this work provides a promising strategy for protecting equipment from multispectral electromagnetic interference and inhibiting infrared detection.

**Supplementary Information:**

The online version contains supplementary material available at 10.1007/s40820-025-01750-z.

## Highlights

- A highly aligned and compact MXene film for ultra‑broadband and ultra-high electromagnetic interference shielding was fabricated by continuous centrifugal spraying strategy.
- The film exhibits an exceptional electromagnetic interference shielding effectiveness (EMI SE) of 45 dB, specific shielding effectiveness of 1.545 × 10^6^ dB cm^2^ g⁻^1^ in gigahertz band (8.2–40 GHz), and EMI SE of 59 dB in terahertz band (0.2–1.6 THz).
- The film exhibits an ultra-low infrared emissivity of 0.1 in the infrared band (2.5–16.0 μm) and an efficient electrothermal performance.

**Supplementary Information:**

The online version contains supplementary material available at 10.1007/s40820-025-01750-z.

## Graphical abstract

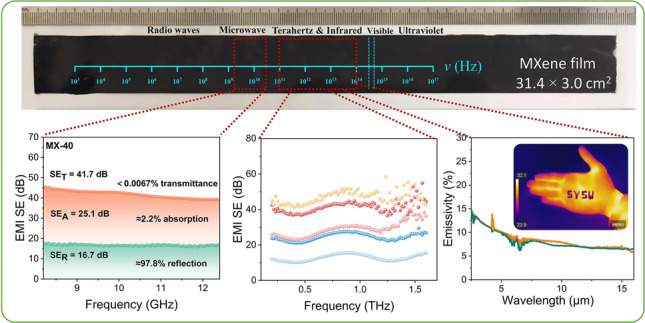

## Introduction

The development of security inspections and aerospace equipment has created an urgent demand for broadband stealth technologies, including those in the gigahertz (GHz), terahertz (THz), infrared (IR), and ultraviolet (UV) bands [1–11]. Recently, numerous stealth materials have been reported. For instance, multi-walled carbon nanotubes (MWCNTs)/silver nanowires (Ag NWs) composite exhibits an electromagnetic interference shielding effectiveness (EMI SE) exceeding 45 dB in the range of 4–40 GHz [12]. Hollow metal-organic frameworks composite films exhibit EMI SE of 66.8 dB in the Ka-band (26.5–40 GHz) and 114.6 dB in the range of 0.1–4.0 THz [13]. CNT films present an EMI SE with an absorption effectiveness ratio of 86.9% and an infrared emissivity value of 0.331 [14]. Ti_3_C_2_T_x_/polyvinyl alcohol (PVA) films can shield nearly 90% UV light [15]. However, different electromagnetic wavebands exhibit distinct shielding mechanisms [16–22]; therefore, necessarily complex structures and materials are generally required to achieve effective shielding and stealth across a wide frequency range. Currently, realizing ultra-broadband electromagnetic shielding performance using a single-material structure remains a significant challenge.

Titanium carbide (Ti_3_C_2_T_x_) MXene, an emerging two-dimensional (2D) transition metal carbide, exhibits low infrared emissivity [23–27], superior electromagnetic wave absorption capacity, and shielding efficiency, primarily attributed to its abundant surface groups and high conductivity [28–30]. Recently, there has been growing interest in assembling MXene flakes into high-performance macroscopic films, driven by their promising applications in EMI shielding [31–36]. To fully leverage the advantages of MXene for electromagnetic shielding, it is crucial to achieve the assembly of MXene nanosheets into films with high orientation. The key strategies for enhancing the orientation degree of MXene films include optimizing the alignment of nanosheets and reinforcing interlayer interactions between nanosheets [37]. For example, MXene-based films achieve an ordered alignment through suction filtration, resulting in an EMI SE of 57.7 dB [38]. The aligned structure MXene/CNF films are fabricated using a blade-coating strategy, exhibiting an electrical conductivity of 46,685 S m^−1^ [39]. MXene-based fiber is subjected to thermal drawing processing, which enhances the alignment of MXene nanosheets, showing an EMI SE of 61 dB at a thickness of 500 μm [40]. The MXene-based composite exhibits an electrical conductivity of 2.2 × 10^4^ S m^−1^, attributed to the intercalation of the polymer between the MXene flakes [41]. However, the manufacture of highly aligned and compact self-assembled films currently remains a significant challenge [42–44]. Recently, a continuous centrifugal casting method has been reported for synthesizing highly aligned and compact 2D nanosheet films [45]. Alignment is achieved by a shear force from velocity differences, while compaction results from a centrifugal force due to high-speed rotation [45–49].

Here, we achieve the preparation of highly conductive and ultra-low infrared emissivity MXene films with high compactness and orderly alignment through continuous centrifugal spraying of MXene dispersion within a rotating tube. The films with an electrical conductivity of 1.03 × 10^6^ S m^−1^ and an orientation degree of 0.954 have been fabricated at a centrifugal force of 1,006 g (with a rotating rate of 3,000 r min^−1^). As a result, an exceptional EMI SE of 45 dB in the GHz band (8.2–40 GHz) and 59 dB in the THz band (0.2–1.6 THz) is achieved at a film thickness of 2.25 μm. Besides, the film demonstrates an exceptionally high specific shielding effectiveness (SSE/t) of 1.545 × 10^6^ dB cm^2^ g⁻^1^ in the THz band, which is higher than that of other reported shielding materials. Additionally, the film exhibits an ultra-low infrared emissivity of 0.1 in the wide-range infrared band (2.5–16.0 μm), demonstrating impressive IR stealth performance for both room/high-temperature equipment and day-/nighttime outdoor environments. Moreover, the film demonstrates efficient electrothermal performance, including a high saturated temperature (over 120 °C at 1.0 V), a high heating rate (4.4 °C s^−1^ at 1.0 V), and a stable and uniform heating distribution, which can be utilized for de-icing applications.

## Experimental Methods

### Preparation of Ti_3_C_2_T_x_ Nanosheets

Ti_3_C_2_T_x_ nanosheets are prepared using conventional etching methods as previously reported [50–52]. A mixed solution of LiF and HCl is utilized to selectively etch the Al layer from the Ti_3_AlC_2_ (MAX) phase. LiF (3.2 g) is firstly added into HCl (40 mL, 9 M) in a Teflon reagent bottle and stirred for 5 min. Subsequently, 2.0 g of Ti_3_AlC_2_ powder is slowly added within 10 min and continually stirred at 35 °C for 24 h. The resulting mixture is centrifuged for 1.0 min at 5,000 r min^−1^, and the supernatant is discarded. The obtained sediment is further washed with deionized water by repeating the above centrifugation until self-delamination of the MXene nanosheets. The neutral dispersion oscillates on a vortex shaker for 10 min to re-disperse the centrifuged sediment uniformly, and then, the dispersion is centrifuged at 10,000 r min^−1^ for 5 min. This shaking and centrifugation procedure is repeated five times. Finally, the resultant dispersion is centrifuged at 3,000 r min^−1^ for 15 min to obtain the colloidal solution for subsequent experiments [53].

### Preparation of MXene Films and Filtered Films

Initially, a 100-micron-thick polyethylene terephthalate (PET) substrate is cleaned with oxygen plasma and the treated PET is attached to the inner wall of a stainless steel tube (inner diameter, 100 mm). The MXene film is subsequently fabricated by the continuous spraying of MXene dispersion (2 mg mL^−1^) onto the substrate within the rotating tube [45]. The dispersion was sprayed out through a needle with an inner diameter of 0.25 mm. The volume of each single spraying is approximately 0.54 mL. The rotating rate of the stainless steel tube is set as 1,000, 1,500, 2,000, 2,500, and 3,000 r min^−1^, and the heating temperature is 45 °C. The film that is sprayed *n* times is defined as an MX-n film. The filtrated film is prepared by the vacuum-assisted filtrating MXene dispersion, and the filtrated film is peeled off from the PES filter membrane.

### Characterization

The micromorphology of the Ti_3_C_2_T_x_ nanosheets and MX-n films is investigated using scanning electron microscopy (FE-SEM, Hitachi, S-4800). X-ray diffraction (XRD; Bruker, D8 ADVANCE) with Cu Kα radiation is used to measure the structures of MX-n films. The chemical bonds and chemical states of the samples of MX-n films are analyzed via Raman spectrometry (HORIBA, LabRAM HR) and X-ray photoelectron spectrometry (XPS; Thermo Scientific, ESCALAB 250Xi). The EMI SE in the GHz band (8.2–40.0 GHz) and THz band (0.2–1.6 THz) are measured in the air using a network analyzer (VNA, Keysight, N5232) and a fiber-coupled THz time-domain spectroscopy system (THz-TDS; BATOP, TDS 1008), respectively. In the THz shielding test, the PET substrate absorption loss of THz electromagnetic wave is first tested and set as a reference value. The actual THz shielding effectiveness of samples is obtained by deducting the reference value from the measured THz shielding results. The absorption of samples in the infrared band is measured using Fourier transform infrared (FTIR) spectrometers equipped with integrating spheres (Bruker, Vertex, 70). The electrical conductivity is measured by a 4-point probes resistivity measurement system (4 probes tech, China), and transmittance in the visible spectrum is measured by a UV–Vis spectrophotometer (Hitachi, U-4100). The thicknesses of the samples are measured by step profiler (KLA instrument, Alpha-step D-300) and atomic force microscopy (Bruker, Dimension Icon). Wide-angle X-ray scattering (WAXS) tests are conducted on the Anton Paar SAXSpoint5.0 System using an incident Cu-Kα X-ray beam parallel to the film plane and striking on the cross-section of the film. To detect the microstructure of MX-n films, a layer of tungsten is deposited on the upper surface of the films and then cut by a focused ion beam (FIB) to provide cross-sections using an FEI Helios NanoLab 600i (using an acceleration voltage of 30 kV and a current of 2.4 nA). Apply direct current power for Joule heating to the sample using a source measure unit (Keithley 2400). The temperatures are determined, and the infrared images are captured by an infrared thermal imager (Hikmicro, K-20). It is essential to emphasize that, in the process of measuring the temperature of electric heating, we utilize electrical tape with an infrared emissivity of 0.95 applied to the surface of the film.

## Results and Discussion

### Fabrication and Characterization of MX-n Films

**Fig. 1:**
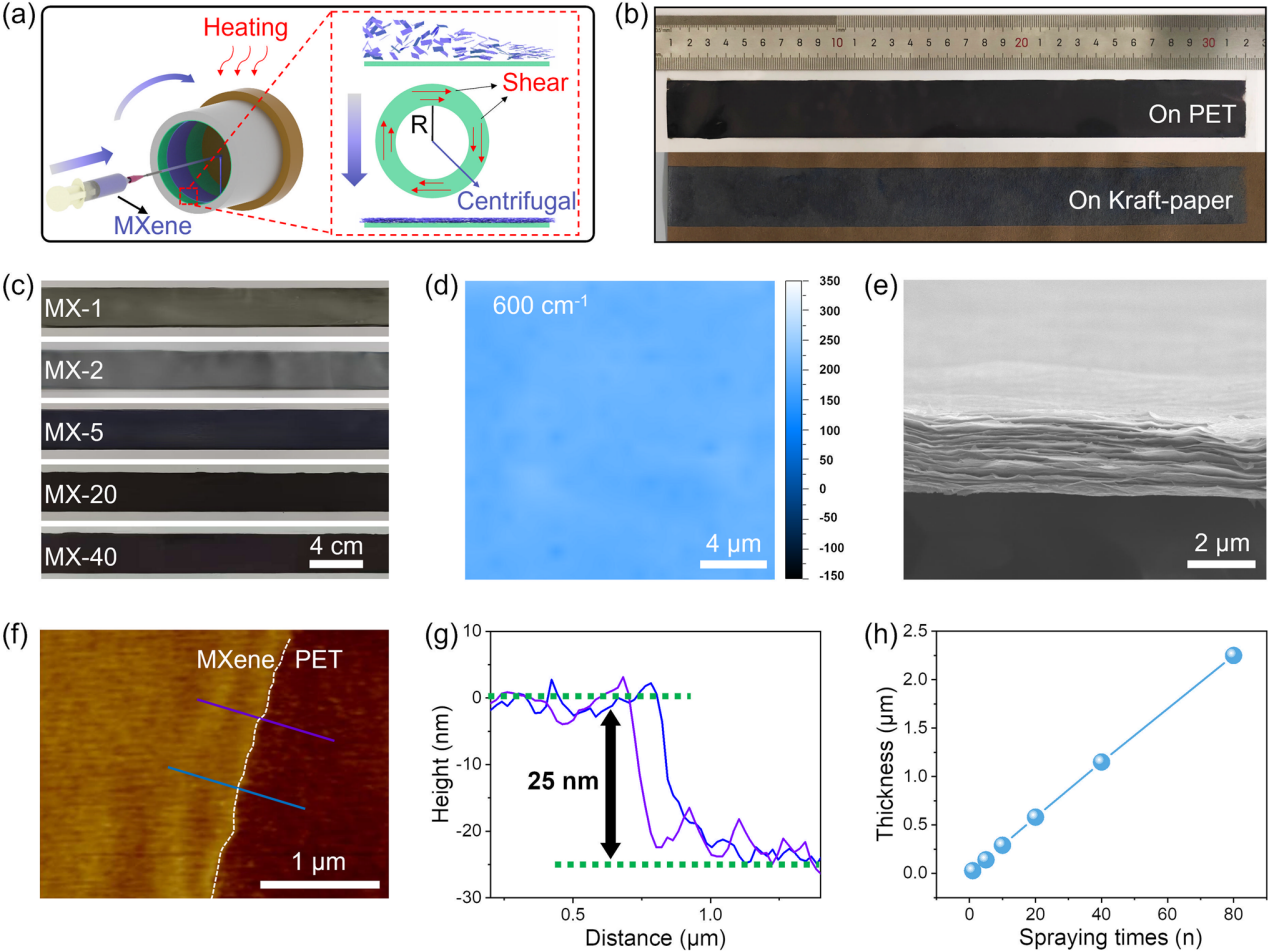
Fabrication and characterization of MX-n films. **a** Schematic of fabrication process for the MXene films. **b** MX-n films deposited on different substrates and **c** MX-n films with different spraying times. **d** Raman mapping of the characteristic mode of the MX-80 film at 600 cm^−1^. **e** Cross-sectional SEM image of the MX-80 film. **f**, **g** AFM image of the MX-1 film. **h** Thickness of MX-n films with different spraying times

The lateral size of the synthesized Ti_3_C_2_T_x_ MXene nanosheets is about 2–5 μm (Fig. S1a). The dispersion of MXene nanosheets is continuously sprayed onto the inner surface of a rotating hollow tube attached to the substrates with low-temperature heating (Fig. 1a). High-speed rotation generates strong centrifugation along the radial direction of the rotating tube. The velocity difference between the rotating tube and sprayed MXene dispersion induces shear force along the tangential direction of the rotating tube. Thus, MXene nanosheets assemble into a highly compact and well-aligned film. All bendable substrates can be attached to the tube and used as substrates for producing MXene films, such as PET, Kraft paper, etc. (Fig. 1b). Uniform and continuous films can be formed on different substrates. The size of the film reaches 31.4 × 3.0 cm^2^, as shown in Fig. 1b. We have prepared a series of films on PET substrate for subsequent analysis. These films are defined as MX-n films, where *n* denotes the times of spraying. As the times of spraying increases, the color of the film gradually darkens, indicating an increase in the thickness of the film (Fig. 1c). From spraying once to 80 times, the film formed on the PET substrate is uniform, continuous, and complete. As shown in Fig. S2, the light transmittance of the film decreases from 50% (MX-1) to nearly 0% as the times of sprayings increases. This indicates that when the times of spraying exceeds 10, the MX-n films almost completely block visible light. The Raman mapping (Figs. 1d and S3) of the characteristic mode at various positions of the MX-80 film and the SEM image of the surface (Fig. S1b) further demonstrate the outstanding uniformity of the as-prepared MXene film. The cross-sectional SEM and FIB-SEM images of the MX-80 film and the MX-40 film show a dense layer structure with almost no interstices inside the films (Figs. 1e and S1c). Meanwhile, the XPS results (Fig. S4) confirmed the successful fabrication of the MXene film with minimal oxidation. The AFM image (Fig. 1f, g) clearly exhibits the MX-1 film with an approximate thickness of 25 nm. As the times of spraying increases, the thickness of the film increases linearly. The thickness of an MX-80 film is approximately 80 times greater than that of the MX-1 film, which suggests significant repeatability in every spraying and controllability in the thickness of the films (Figs. 1h and S5). MX-n films with varying thicknesses (Fig. S7) exhibit similar electrical conductivities, indicating a stable and controllable fabrication process.

**Fig. 2:**
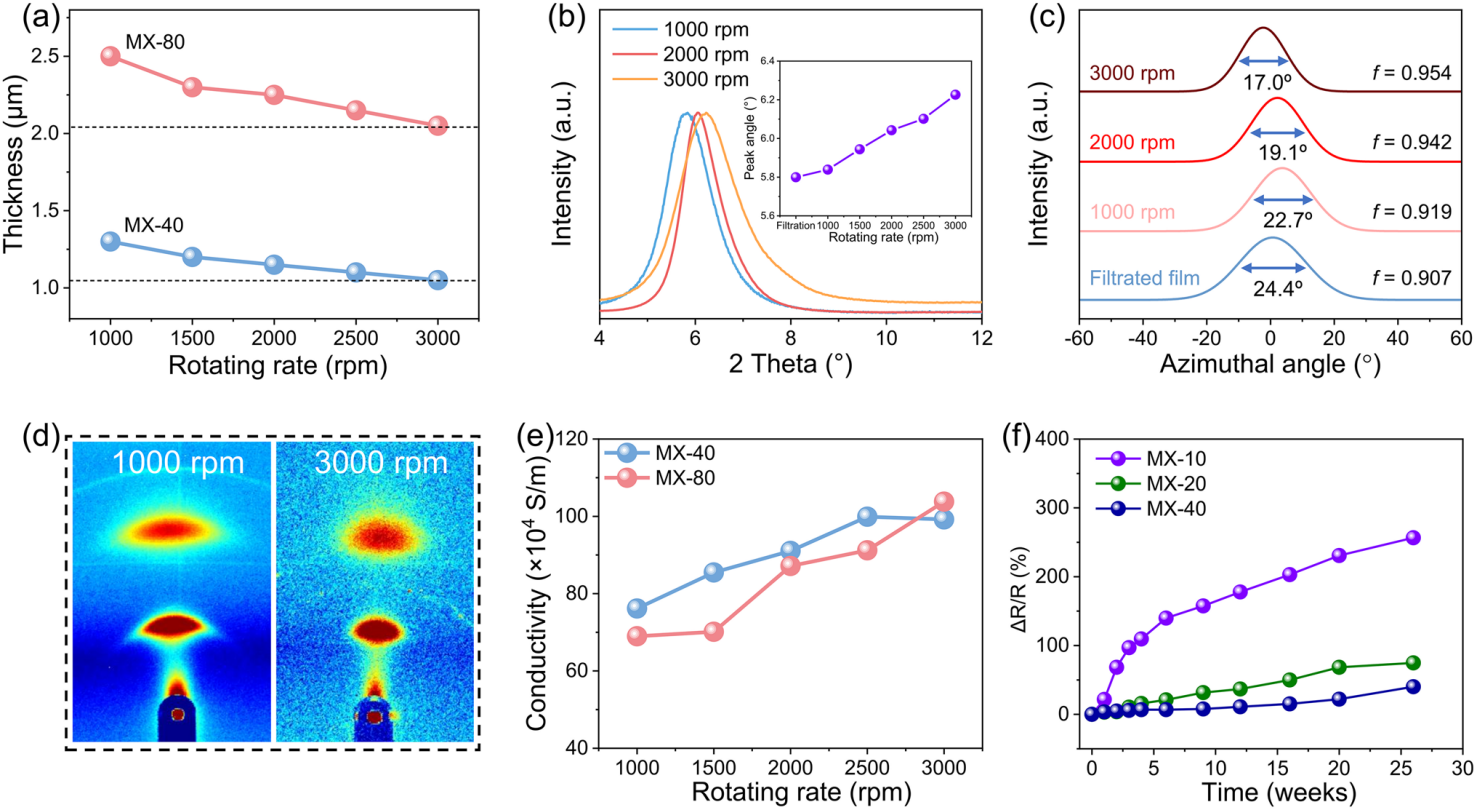
Structural and electrical properties of MX-n films. **a** Thickness of MX-40 films and MX-80 films prepared at different rotating rates. **b** XRD patterns of MX-n films prepared at different rotating rates. **c** Corresponding azimuthal scan profiles for the (002) peak and **d** WAXS patterns for an incident Cu-Kα X-ray beam parallel to the film plane for MX-n films prepared at different rotating rates. **e** Conductivity of MX-40 films and MX-80 films prepared at different rotating rates. **f** Variation in sheet resistance of MX-n films exposed to air over time

The compactness of MXene nanosheets is determined by the centrifugation. Centrifugal force during the assembly of MXene nanosheets can be tuned by adjusting the rotating rate of the tube. When the rotating rate of the tube is set at 1,000 r min^−1^, the calculated centrifugal force is 112 g, while the centrifugal force increases to 1,006 g at 3,000 r min^−1^, almost 9 times increase. Meanwhile, the velocity difference between the tube and MXene dispersion also improves the alignment of MXene nanosheets. The reduction in film thickness confirms the reduction in interlayer spacing between the MXene nanosheets with the increases rotating rate (Figs. 2a and S6). When the rotational rate increases from 1,000 to 3,000 r min^−1^, the thickness of the MX-40 film decreased from 1.30 to 1.05 μm, corresponding to a reduction of approximately 19.2%. The decrease in thickness indicates an increase in the compact of the film. XRD and WAXS techniques are employed to assess the improvement in the compactness and alignment of MX-n films. Based on Bragg’s law, $2dsin\theta =\lambda$, the position of the XRD peak (θ) determines the interlayer distance (*d*) of the films, which is correlated with the alignment and compactness of the film. Figure 2b shows the XRD patterns of MX-80 films prepared at different rotating rates. It is evident that the (002) peak position of MX-n films upshifts from 5.8° to 6.2° when the rotating rate is increased from 1,000 to 3,000 r min^−1^, confirming that more compact MXene film is achieved. WAXS pattern (Figs. 2c, d and S8) demonstrated a higher degree of alignment at 3,000 r min^−1^ compared with a lower rotating rate. Here, the Hermann orientation factor (HOF) is used to evaluate the arrangement order between nanosheets. When the HOF value is 0, it indicates complete disorder, and when the value is 1, it indicates a complete alignment arrangement. Specifically, as the rotating rate increases, the HOF of the film rises from 0.919 to 0.954, and the full width at half maximum (FWHM) decreases from 22.7° to 17.0°. Highly compact and orderly alignment of MXene nanosheets also results in improved electrical conductivity. When the rotating rate increases from 1,000 to 3,000 r min^−1^ (Fig. 2e), the conductivity of MX-n films increases by approximately 50% at the higher rotating rate, ultimately reaching a maximum value of 1.03 × 10^6^ S m^−1^ at 3,000 r min^−1^. In addition, after 26 weeks of exposure to the air at room temperature (20 °C), the sheet resistance of the MX-40 film only increased by 40% (Fig. 2f), indicating its excellent oxidation resistance and high degree of orientation.

### EMI Shielding Performances of MX-n Films

**Fig. 3:**
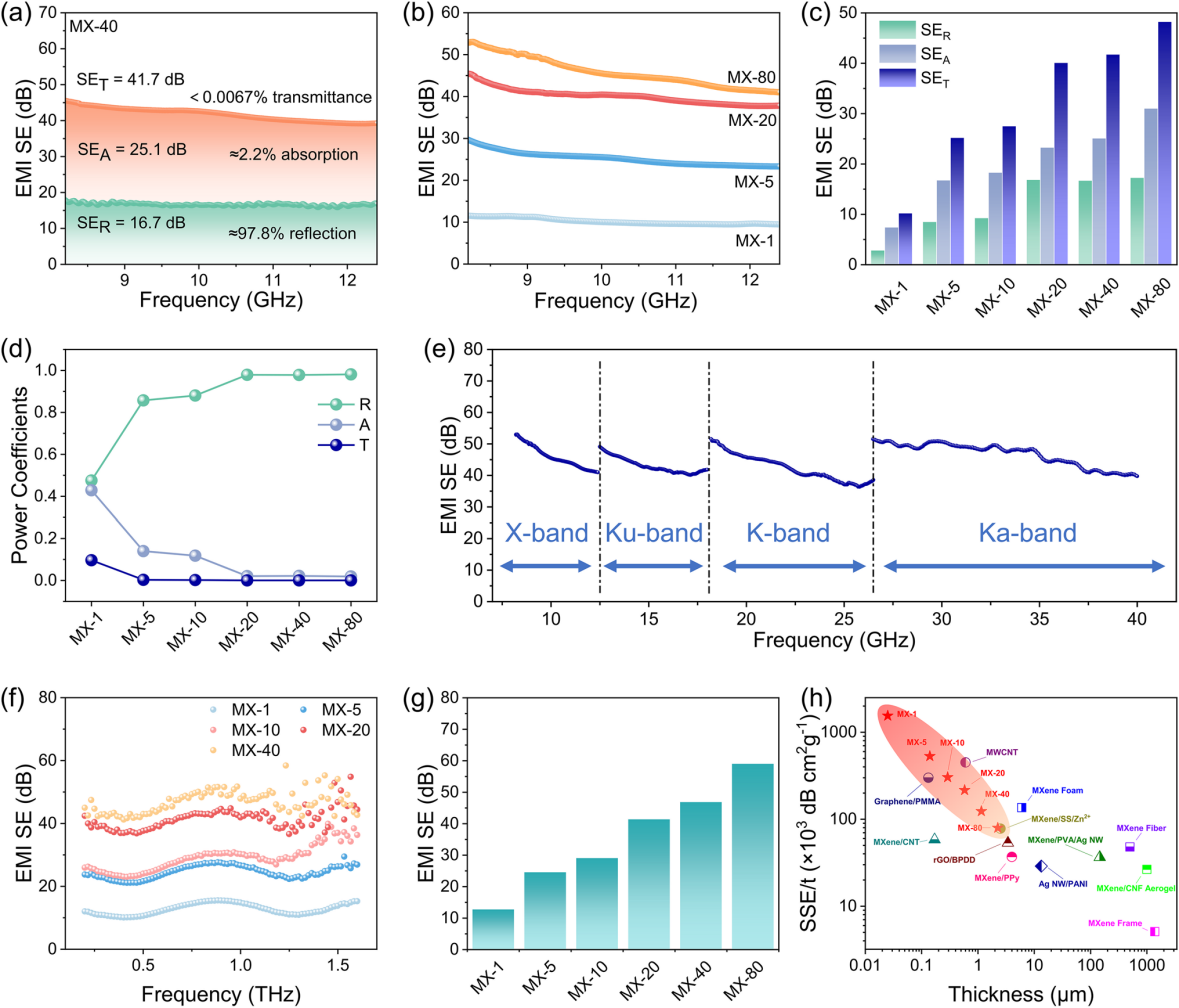
EMI shielding performance of MX-n films. **a** Contributions of SE_R_ and SE_A_ to SE_T_ for the MX-40 film. **b** Total EMI SE of MX-n films with different spraying times in the X-band (8.2–12.4 GHz). **c** Average refection (SE_R_), absorption (SE_A_), and total EMI SE (SE_T_) of the MX-n films with different spraying times in the X-band. **d** Power coefficients of MX-n films with different spraying times. **e** Total EMI SE of an MX-80 film in the frequency range of 8.2–40.0 GHz. **f** Total EMI SE and **g** average total EMI SE of MX-n films with different spraying times in the frequency range of 0.2–1.6 THz at 2,000 r min^−1^. **h** Comparison of the SSE/t versus thickness in MX-n films and other shielding materials (detailed data thereof are listed in Table S1)

To investigate the effects of thickness and rotating rate on the EMI shielding performances of MX-n films, we have fabricated MX-n films with different spraying times and rotating rates. The average total EMI SE (SE_T_) of an MX-40 film (~ 1.15 μm) is 42 dB in the X-band, including 17 dB of refection (SE_R_) and 25 dB of absorption (SE_A_) (Fig. 3a). The increased spraying times of MX-n films results in the enhanced EMI shielding performance (Fig. 3b, c). As the spraying times increases, the average SE_R_ in the X-band rises from 3 to 17 dB, while the average SE_A_ increases from 7 to 31 dB. At a thickness of 2.25 μm, the MX-80 film achieves an average EMI SE of 48 dB in the range of 8.2–12.4 GHz and 45 dB in the range of 8.2–40 GHz. The attenuation of EM intensity results from the reflection and absorption mechanisms of electromagnetic waves by shielding materials [54]. The electromagnetic shielding mechanism of MX-n films primarily involves reflection, which depends on the impedance mismatch at the interface between two media with different impedances. Elevated film conductivity may lead to significant impedance mismatch, while the introduction of additional layer structures with increased thickness can enhance multiple reflections, thereby improving the overall reflection and absorption of the film. Figure 3d clearly illustrates the ratios of reflection and absorption in electromagnetic shielding effectiveness. As the spraying times increases, the reflection ratio exceeds 98%. Meanwhile, the MX-80 film also exhibits excellent electromagnetic shielding performance in the 8.2- to 40.0-GHz frequency band (Fig. 3e).

MX-n films also demonstrate excellent electromagnetic shielding performance in the frequency ranges of 0.2–1.6 THz (Fig. 3f, g). In particular, the MX-80 film achieves an average EMI SE of 59 dB in the range of 0.2–1.6 THz. The rotating rate of MX-n films is another key factor influencing their EMI SE. Interestingly, the total EMI SE of MX-n films exhibits minimal variation with changes at different rotating rates (Fig. S9). The primary reason for this is that as the rotating rate increases, the film thickness decreases while conductivity increases. Significantly, EMI shielding performance is determined by electrical conductivity and film thickness simultaneously [54]. High electrical conductivity and high film thickness tend to high EMI shielding performance.

Thicker films can provide adequate shielding but at the expense of both gained weight and increased space occupation in the EMI shielding system. To evaluate the EMI shielding performance *per* unit thickness, we calculated the absolute shielding effectiveness (EMI SE/t, measured in dB μm^−1^) by dividing the EMI SE by the thickness. The column charts presented in Figs. S9c, f and S11c show that EMI SE/t significantly increases with the increased rotating rates in both the X-band and the THz band. This suggests the remarkable superiority of MX-n films in electromagnetic shielding *per* unit thickness under high rotating rates. For the MX-1 film, its SE/t can reach an astonishing 400 dB μm^−1^ in the X-band (8.2–12.4 GHz) and 500 dB μm^−1^ in the frequency range of 0.2–1.6 THz (Fig. S10). We also calculate the ratio of EMI SE to film density and thickness to derive an additional performance indicator for the material (SSE/t, measured in dB cm^2^ g^−1^) (Table S1). The data in the table clearly show that our films exhibit high SSE/t values, reaching 1.545 × 10^6^ dB cm^2^ g⁻^1^. Furthermore, we have also compared the MX-n films with other reported electromagnetic shielding samples (Fig. 3h). It is evident that the MX-1 film exhibits an outstanding SSE/t.

### Infrared Stealth and Electrothermal Properties of MX-n Films

**Fig. 4:**
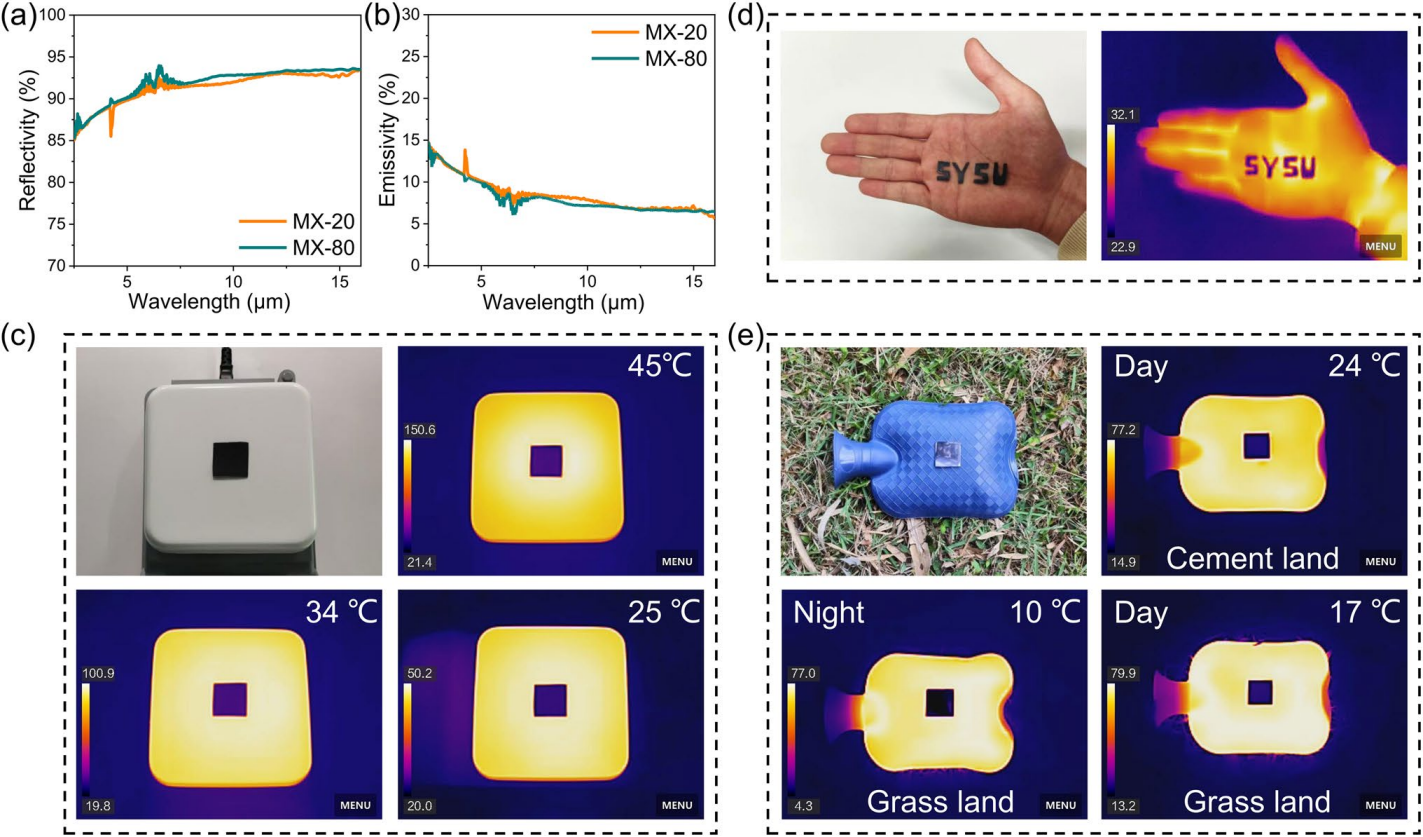
**a** IR reflectivity curves and **b** IR emissivity curves of MX-n films. **c** IR images and optical image of an MX-80 film on the thermal stages of 150, 100, and 50 ºC. **d** IR images and optical image of an MX-80 film on palm. **e** IR images and optical image of the MX-80 film in different daytime and nighttime outdoor environments

All objects with a temperature exceeding absolute zero continuously emit thermal radiation [55]. Notably, the thermal radiation produced at elevated temperatures during the operation of equipment can lead to substantial levels of radiation, which can be easily identified by infrared detectors. Therefore, it is crucial to develop and manufacture high-performance infrared stealth materials that efficiently mitigate the infrared radiation emitted by these objects. Infrared detectors identify targets by detecting the radiation difference between the target and its background. When a target has low emissivity, its emitted radiation signal becomes indistinguishable from that of the background, thereby achieving infrared stealth. To investigate the infrared stealth properties of MX-n films, the infrared reflectance of the films was measured. Figure 4a demonstrates that the MX-n films exhibit an average infrared reflectivity ranging from approximately 85% to 90% as the spraying times increases. This behavior is analogous to the reflectivity observed in metals [56, 57]. According to Kirchhoff’s radiation law, the infrared emissivity of MX-n films can be calculated (Fig. 4b). MX-n films exhibit a low infrared emissivity of 0.1 within the wavelength range of 2.5–16.0 μm, suggesting their significant potential for applications in infrared stealth.

To evaluate the infrared stealth performance, an MX-80 film is positioned on a thermal stage with different temperatures (50, 100, and 150 °C). As infrared images illustrated in Fig. 4c, the MX-80 film is clearly cooler than the thermal stage at different temperatures. The area covered by the MX-80 film is effectively camouflaged in the infrared image, closely resembling the dark blue color of the background. It is noteworthy that the corresponding infrared stealth performance can be sustained at the thermal stage with a higher temperature of 150 °C. This indicates that the MX-80 film can effectively inhibit thermal radiation from high-temperature sources. Furthermore, as depicted in Fig. 4d, the MX-80 film (scissored into the shape of English letters) provides effective thermal camouflage for the human body (specifically the palm). In comparison with the surrounding environment, the MX-80 film displays a darker blue hue and a reduced temperature. It is important to acknowledge that the application scenarios for infrared stealth materials may vary in practical contexts, such as during both daytime and nighttime conditions. Notably, there are substantial differences in the infrared radiation emitted by high-temperature heat sources and their backgrounds across these different scenarios. Consequently, it is essential for infrared stealth materials to possess strong environmental compatibility in order to adapt to variations in the background effectively. The MX-80 film is specifically positioned on the grass to evaluate its infrared stealth performance under both day and night conditions. As illustrated in Fig. 4e, the film demonstrates exceptional infrared stealth capabilities across various environments, including cement surfaces and grasslands, during both day and night.

**Fig. 5:**
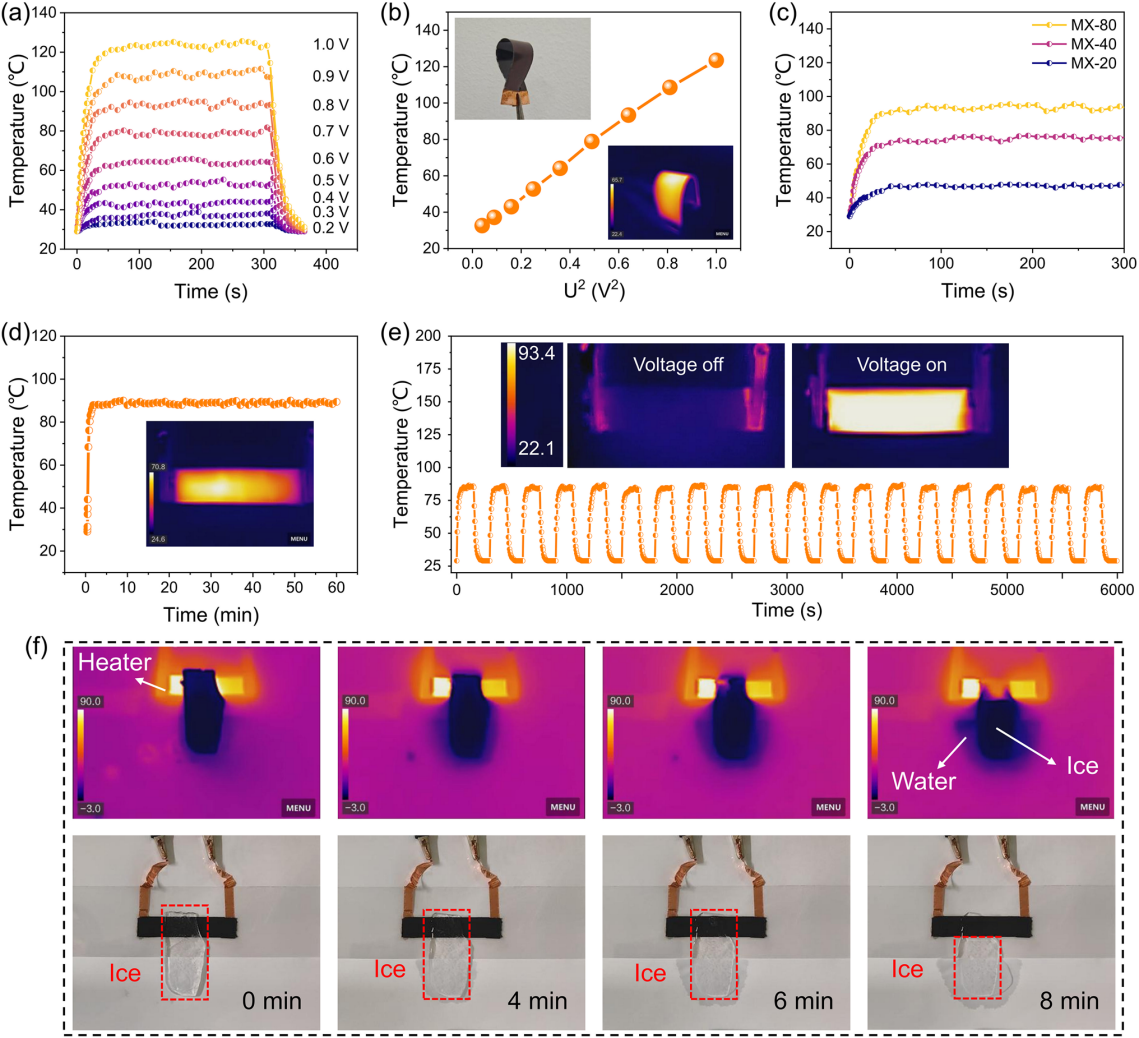
Electrothermal properties of MX-n films. **a** Time-dependent surface temperatures and **b** average surface temperatures of the MX-80 film under different supplied voltages. Insets show the flexibility of the MX-80 film. **c** Time-dependent surface temperature of MX-n films with different spraying times at 0.8 V supplied voltage. **d** Temperature durability of the MX-80 film heater in 60 min under continuous 0.8 V voltage applied. Inset shows uniform heating of the MX-80 film heater. **e** Thermal response of the MX-80 film heater under cyclic voltage on–off at 0.8 V. Insets demonstrate the on and off statuses of the MX-80 film. **f** De-icing images of the MX-80 film heater

The MX-n films, characterized by their exceptional electrical conductivity and density, have created opportunities for the development of high-performance electric heating devices, including electric heaters and de-icing systems. According to Joule’s Law, $J={U}^{2}{R}^{-1}T$, where *J* is the heat generated by the Joule effect, *U* is the applied voltage, *R* is the resistance of the heaters (MX-n films), and *T* is the time. Thus, the heat generated is directly correlated with the applied voltage and resistance. As shown in Fig. 5a, the highest saturation temperature of the films increases from 33 to 123 ºC as the applied voltages increase from 0.1 to 1.0 V. The corresponding current values of the MX-n films at different supplied voltages are shown in Fig. S12a. The temperature data are taken while the heating film is freestanding in the air, and the covering area of the heating film is 2.0 × 1.0 cm^2^. The MX-80 film possesses a relatively fast heating-up time, and the MX-80 film rises from room temperature to saturation temperature within 30 s at different applied voltages (4.4 °C s^−1^ at 1.0 V). It is worth noting that, as illustrated in Fig. 5b, the saturation temperature exhibits a linear increase with the elevation of *U*^2^, suggesting that the MX-80 film possesses stable linear electric heating performance. The illustration in the inset of Fig. 5b further demonstrates that the MX-80 film maintains uniform thermal performance even when subjected to bending. According to Joule’s law, a lower resistance allows for greater heat conversion at a given applied voltage. The electrothermal properties of MX-n films with different spraying times are investigated in Figs. 5c and S12b. At an applied voltage of 0.8 V, the saturation temperatures of the MX-n films are observed to be 47, 75, and 90 °C for films with 20, 40, and 80 spraying times, respectively. Consequently, an increase in the spray times of MX-n films can enhance the saturation temperature of the heating film.

The heater durability is assessed by applying continuous voltage (0.8 V) to the MX-80 film (Fig. 5d). The saturation temperature of the MX-80 film can remain at about 90 °C for 1 h, demonstrating excellent durability for long-term stable heaters. At an applied voltage of 0.8 V, the heater demonstrates periodic stability in saturation temperature during the voltage on and off cycles, as illustrated in Fig. 5e. The MX-80 film heater is employed to illustrate the electric heating efficiency. As depicted in Fig. 5f, an ice approximately 5 mm in thickness is positioned above the heater, with a transparent PET film interposed between the heater and the ice to avoid wetting of the MX-80 film. The infrared camera effectively captured the ice-melting process. Initially, the ice remained intact. However, after 8 min of heating, the portion above the MX-80 film heater has almost completely transitioned to water, while the section not subjected to the MX-80 film heater remained unchanged. This suggests that the MX-80 film heater proposed in this work is effective for de-icing applications. Therefore, MX-n films hold vast potential when utilized as an electric heater.

## Conclusion

In summary, we report the preparation of highly conductive and ultra-low infrared emissivity MXene films with high compactness and orderly alignment, suitable for ultra-broadband electromagnetic shielding and infrared stealth. The MXene films are achieved through continuous centrifugal spraying of MXene dispersion within a rotating tube. An exceptional EMI SE of 45 dB in the GHz band (8.2–40 GHz) and 59 dB in the THz band (0.2–1.6 THz) is achieved when the thickness of the MXene film amounts to 2.25 μm. Besides, the MXene film demonstrates an exceptionally high SSE/t of 1.545 × 10^6^ dB cm^2^ g⁻^1^ in the THz band, which is higher than that of other reported shielding materials. Additionally, the MXene film exhibits an ultra-low infrared emissivity of 0.1 in the infrared band (2.5–16 μm), which is highly suitable for infrared stealth applications. Moreover, the film demonstrates efficient electrothermal performance, including a high saturated temperature (over 120 °C at 1.0 V), a high heating rate (4.4 °C s^−1^ at 1.0 V), and a stable and uniform heating distribution, which can be utilized for de-icing applications. Consequently, the fabricated MXene films demonstrate high potential for practical multispectral electromagnetic shielding and all-day outdoor infrared stealth performance in military and civilian fields.

## Supplementary Information

Below is the link to the electronic supplementary material.

Supplementary file1 (PDF 3123 KB)
