# Supplementary material for: Ultra‑Broadband and Ultra-High Electromagnetic Interference Shielding Performance of Aligned and Compact MXene Films
Source: Nanomicro Lett. 2025 Apr 27;17:234. doi: 10.1007/s40820-025-01750-z (PMC12034605; doi:10.1007/s40820-025-01750-z)
Supplement: Supplementary file 1 — Supplementary file1 (PDF 3123 KB) [file 40820_2025_1750_MOESM1_ESM.pdf]

Supporting Information

Ultra-Broadband, and Ultra-high Electromagnetic Interference Shielding Performance of Aligned and Compact MXene Films

Weiqiang Huang[1#], Xuebin Liu[1#], Yunfan Wang[1], Jiyong Feng[1], Junhua Huang[1], Zhenxi Dai[1], Shaodian Yang[1,2], Songfeng Pei[3,4], Jing Zhong[5], and Xuchun Gui[*1]

[1]*State Key Laboratory of Optoelectronic Materials and Technologies, School of Electronics and Information Technology, Sun Yat-sen University, Guangzhou 510275, Guangdong, China*

[2]*National Key Laboratory of Materials for Integrated Circuits, Shenzhen Institute of Advanced Electronic Materials, Shenzhen Institute of Advanced Technology, Chinese Academy of Sciences, Shenzhen 518055, China*

[3]*Shenyang National Laboratory for Materials Science, Institute of Metal Research, Chinese Academy of Sciences, 72 Wenhua Road, Shenyang 110016, P. R. China*

[4]*School of Materials Science and Engineering, University of Science and Technology of China, 72 Wenhua Road, Shenyang 110016, P. R. China*

[5]*Key Lab of Structure Dynamic Behavior and Control (Harbin Institute of Technology), Ministry of Education, Harbin, 150090, Heilongjiang, China*

[#] *These authors contributed equally.*

*To whom correspondence. E-mail: *guixch@mail.sysu.edu.cn*

Supporting Method S1. Theoretical calculation of the centrifugal force.

The numerical value of the relative centrifugal force is determined using the following formula [1]:

$$G = \frac{R \times \omega^2}{g} = \frac{R \times \pi^2 \times S^2}{g \times 30^2} \tag{1}$$

Where *G* is the relative centrifugal force (denoted in units of gravitational acceleration, *g*), *g* is the gravitational acceleration (m s^{-2}), *R* is the centrifugal radius (m), ω is the angular velocity (rad s^{-1}), and *S* is the rotating rate (rpm). The specific data of relative centrifugal force are presented as follows in the table.

Centrifugal radius (m),	Rotating rate (rpm)	Relative centrifugal force (*g*)
0.1	1000	112
	1500	252
	2000	447
	2500	699
	3000	1006

Supporting Method S2. WAXS and theoretical calculation of Herman's orientation factor.

Wide-Angle X-ray Scattering (WAXS) tests are conducted on the Anton Paar SAXSpoint5.0 System using an incident Cu-Kα X-ray beam parallel to the film plane and striking on the cross-section of the film. The distance between the sample and the detector is 13.25 cm. The samples for WAXS tests were 1.5-mm-wide, 10-mm-long strips. The alignment degree of MXene flakes

is quantified using Herman's orientation factor (*f*), which is defined by the following formula [2,3]:

$$f = \frac{3}{2}\frac{\int_0^{\pi/2} I(\varphi)\cos^2(\varphi)\sin(\varphi)\,d\varphi}{\int_0^{\pi/2} I(\varphi)\sin(\varphi)\,d\varphi} - \frac{1}{2} \tag{2}$$

Where *I(φ)* is the scattering intensity integrated along the azimuthal direction centered at the (002) signal of MXene films. The numerical values are obtained from a Gauss function that is fitted to the data (Figure S7).

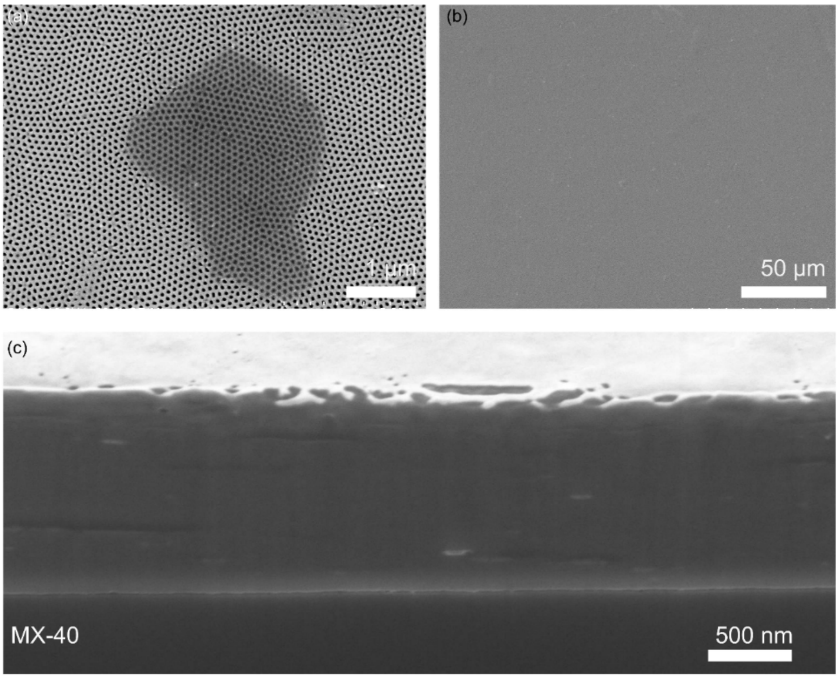

Fig. S1 a SEM image of an MXene flake. **b** SEM image of an MX-80 film. **c** Cross-sectional SEM image of an MX-40 film.

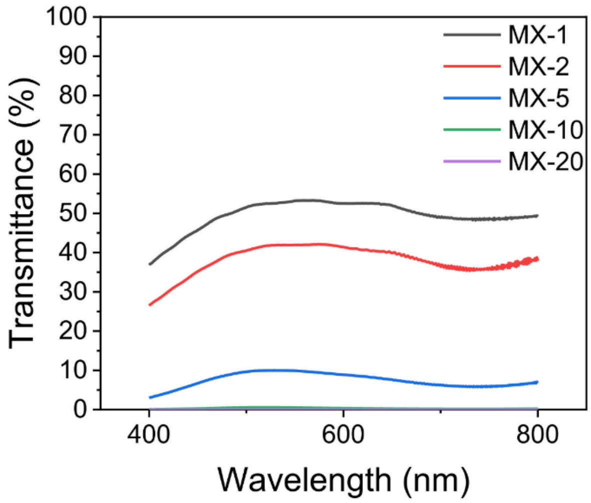

Fig. S2 The light transmittance of the MX-n films with different spraying times.

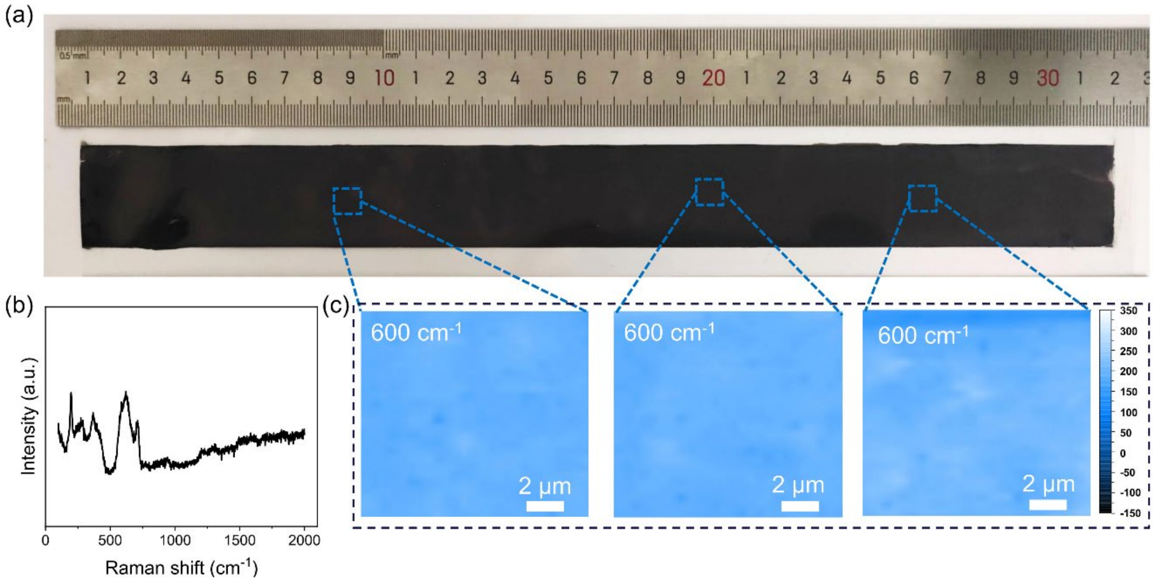

Fig. S3 **a** The MX-80 film deposited on PET substrate. **b** Raman spectra of the MX-80 film. **c** The Raman mapping of the characteristic mode at different positions of the MX-80 film at 600 cm^{-1}

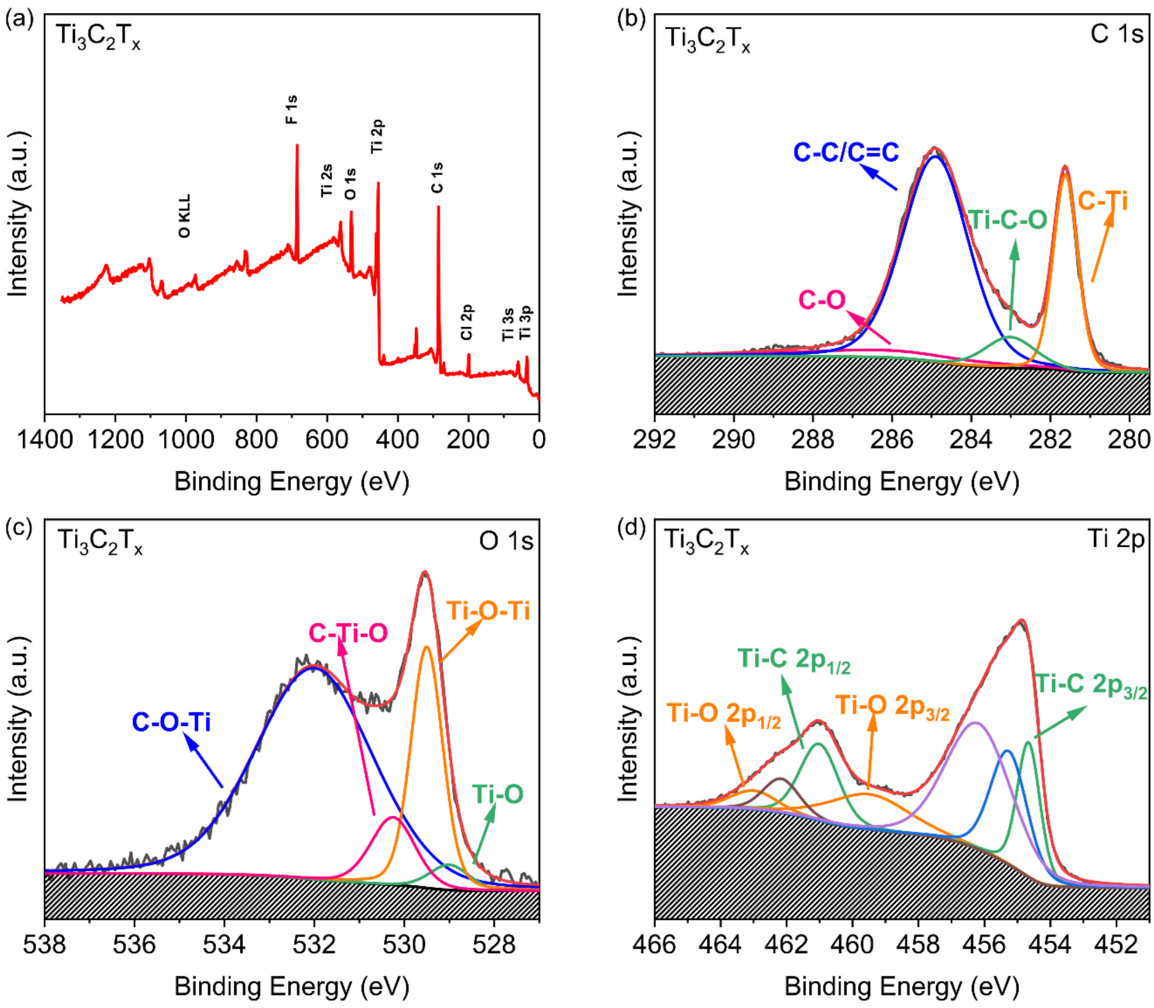

Fig. S4 **a** XPS spectra of the MX-80 film. **b** C 1s, **c** O 1s, and **d** Ti 2p spectra of the MX-80 film. The XPS results confirm the successful fabrication of $Ti_3C_2T_x$ MXene films [4], with no evidence of significant oxidation.

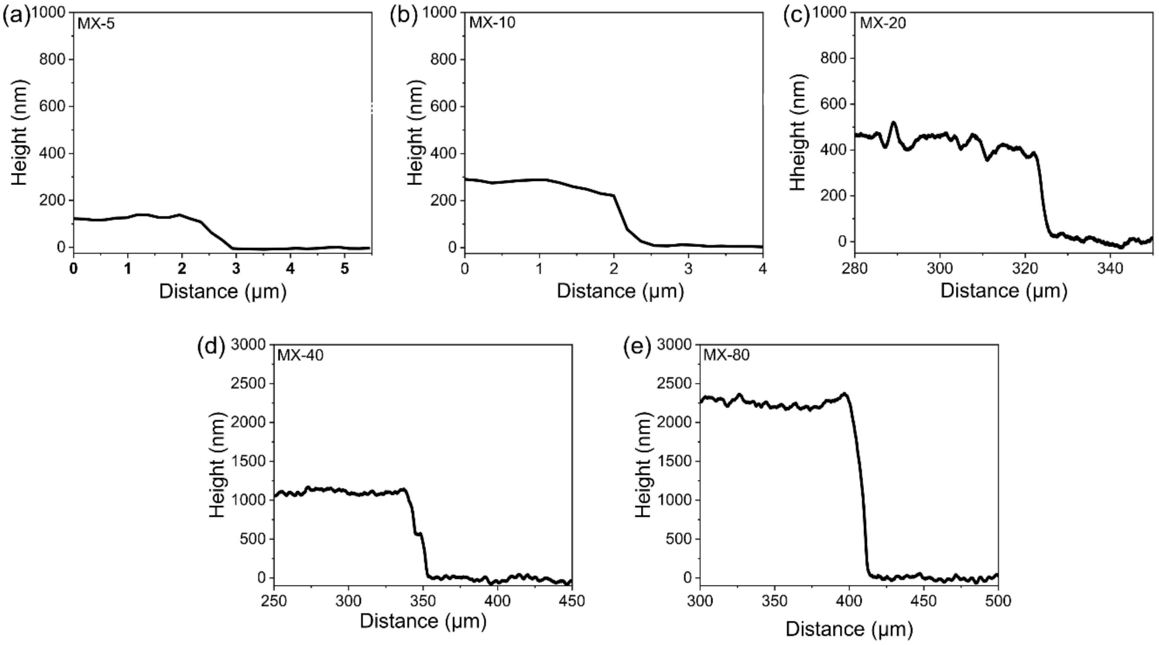

Fig. S5 Thickness distribution of **a** MX-5 film and **b** MX-10 film measured by AFM. Thickness distribution of **c** MX-20 film, **d** MX-40 film, and **e** MX-80 film measured by step profiler.

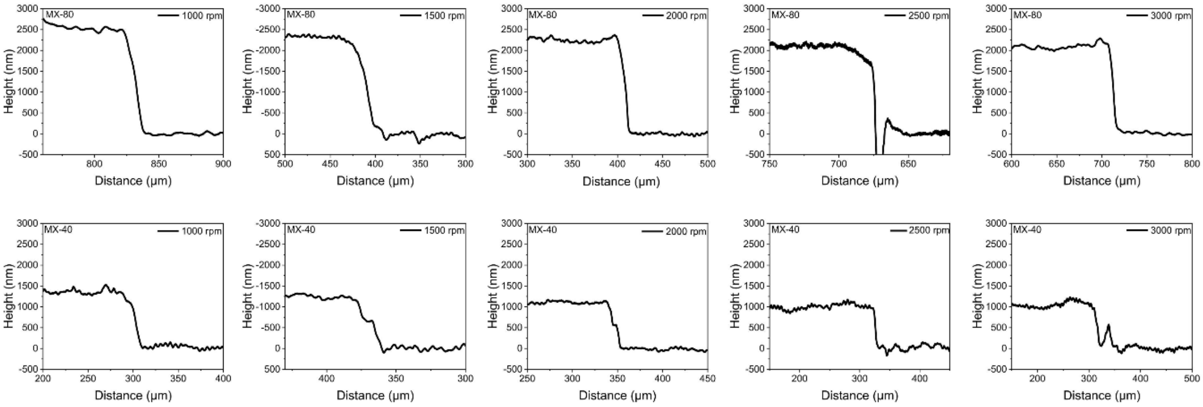

Fig. S6 Thickness distribution of MX-40 films and MX-80 films at different rotating rates measured by step profiler.

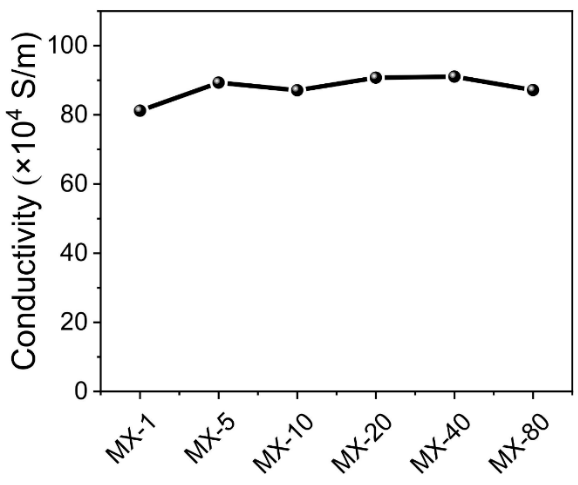

Fig. S7 Conductivity of MX-n films with different spray times.

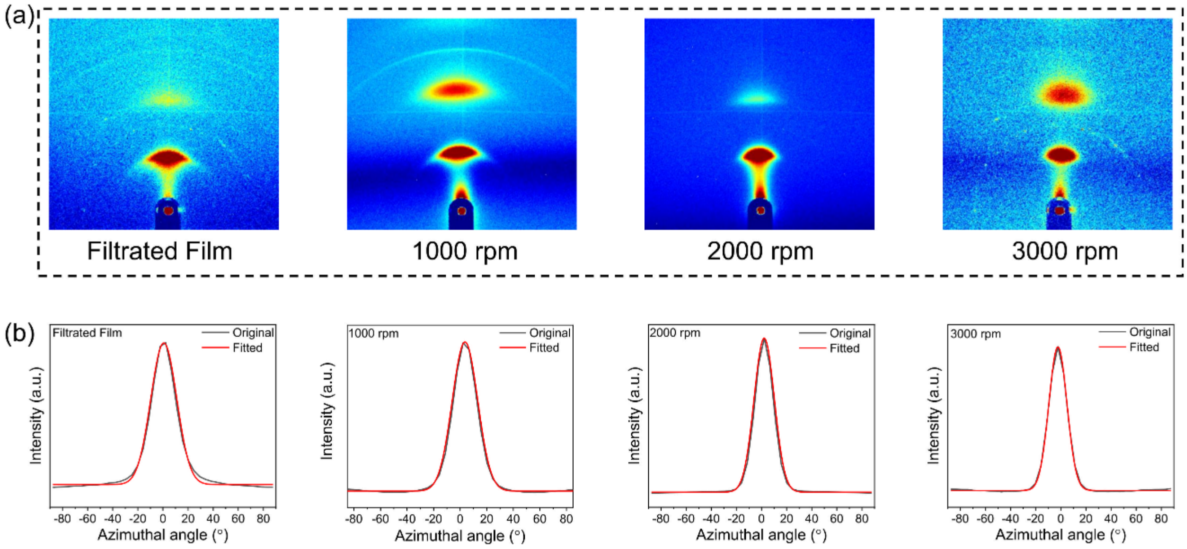

Fig. S8 a WAXS patterns for an incident Cu-Kα X-ray beam parallel to the film plane and **b** **c**orresponding azimuthal scan profiles for the 002 peak for MX-n films and filtrated film.

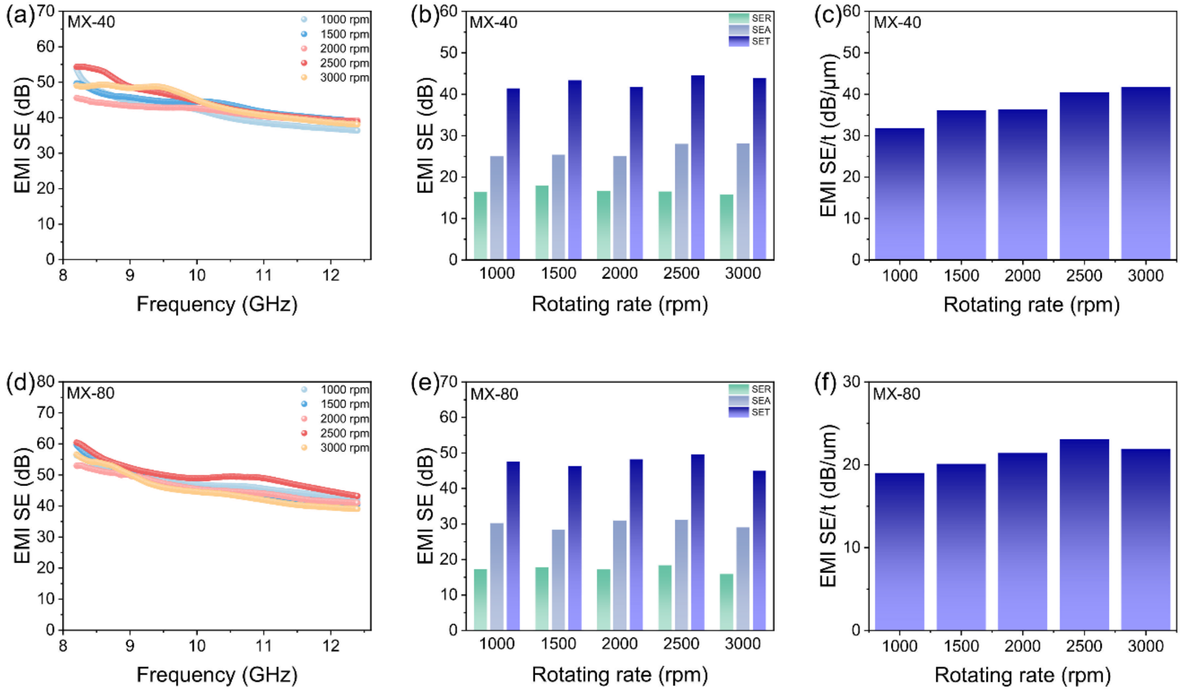

Fig. S9 Total EMI SE of MX-40 films **a** and MX-80 films **d** at different rotating rates in the X-band. Average refection (SE_R), absorption (SE_A), and total EMI SE (SE_T) of the **b** MX-40 films and **e** MX-80 films at different rotating rates in the X-band. EMI SE/t of **c** MX-40 films and **f** MX-80 films at different rotating rates in the X-band.

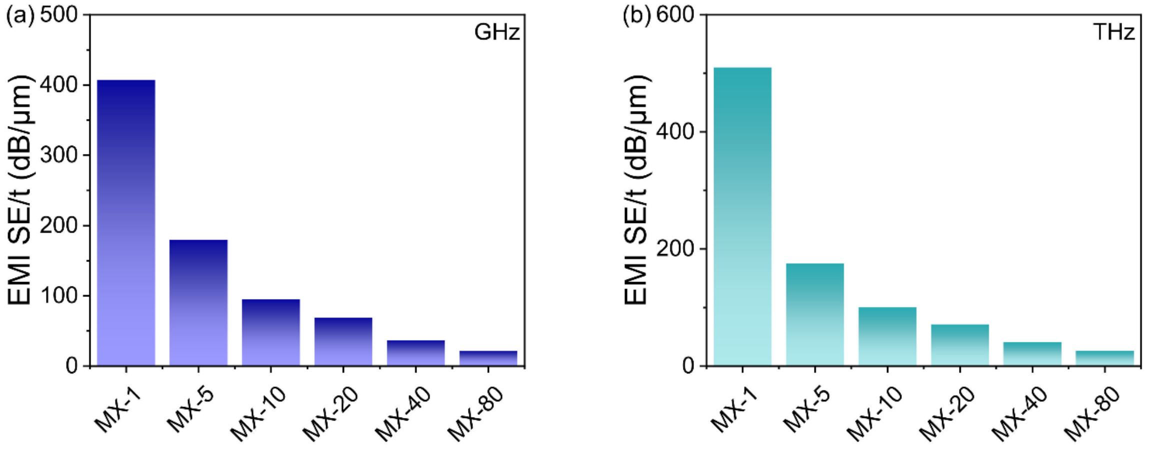

Fig. S10 EMI SE/t of MX-n films with different spraying times in **a** the X-band (8.2-12.4 GHz) and **b** the frequency range of 0.2-1.6 THz.

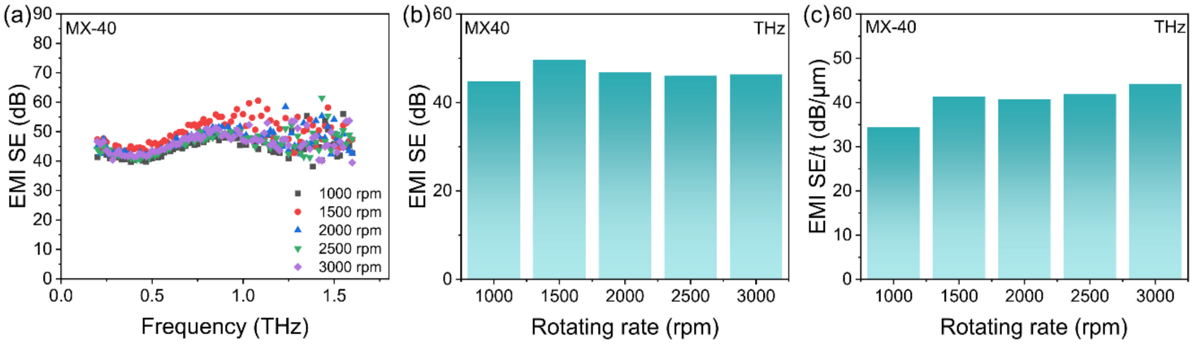

Fig. S11 a Total EMI SE of MX-40 films at different rotating rates in the frequency range of 0.2-1.6 THz. **b** Average EMI SE of the MX-40 films at different rotating rates in the frequency range of 0.2-1.6 THz. **c** EMI SE/t of MX-40 films at different rotating rates in the frequency range of 0.2-1.6 THz.

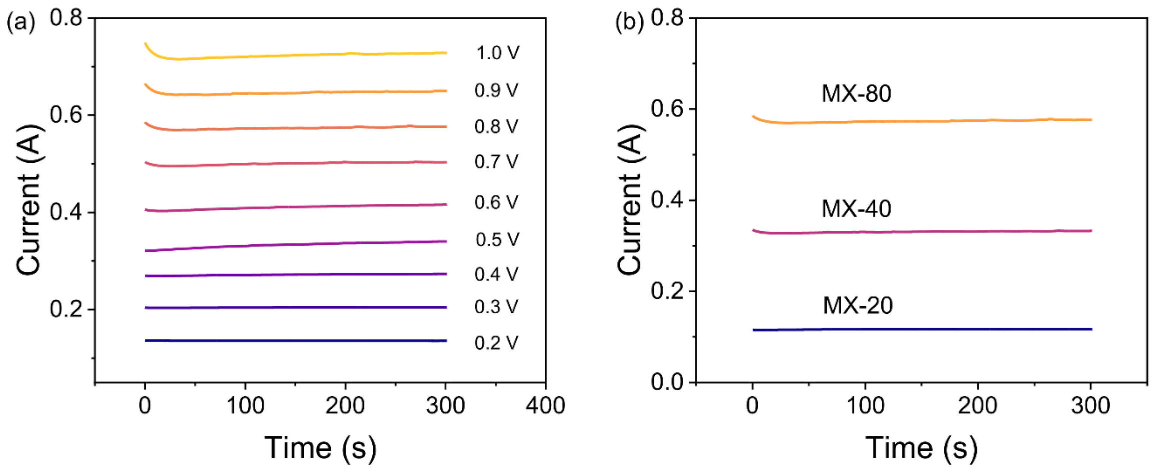

Fig. S12 a Corresponding current values of MX-n films at different supplied voltages. **b** Corresponding current values of the MX-n films with different spraying times at 0.8 V supplied voltage.

Table S1 Thickness and SSE/t (defined as the SE divided by thickness and density) of various shielding materials.

Sample	Thickness (μm)	SSE/t ($\times 10^3$ dB cm^{2}g^{-1})	Refs.
MXene/CNF Aerogel	1000	26.5	[5]
MXene Foam	6	136	[6]
MXene Fiber	500	48	[7]
MXene Frame	1380	5.1	[8]
MXene/PPy	4	36.9	[9]
MXene/SS/Zn^{2+}	2.52	78	[10]
Graphene/PMMA	0.132	300	[11]
MWCNT	0.6	450	[12]
rGO/BPDD	3.4	53.4	[13]
MXene/PVA/Ag NW	145.4	36.5	[14]
MXene/CNT	0.17	58.2	[15]
Ag NW/PANI	13.3	28.9	[16]
**MX-1**	**0.025**	**1545**	**This work**
**MX-5**	**0.14**	**531**	
**MX-10**	**0.29**	**304**	
**MX-20**	**0.58**	**216**	
**MX-40**	**1.15**	**123.5**	
**MX-80**	**2.25**	**79.5**	

Supplementary References

[1] J. Zhong, W. Sun, Q. Wei, X. Qian, H.-M. Cheng, et al., Efficient and scalable synthesis of highly aligned and compact two-dimensional nanosheet films with record performances. Nat. Commun. **9**, 3484 (2018). https://doi.org/10.1038/s41467-018-05723-2.

[2] H. Park, K. H. Lee, Y. B. Kim, S. B. Ambade, S. H. Noh, et al., Dynamic assembly of liquid crystalline graphene oxide gel fibers for ion transport. Sci. Adv. **4**, eaau2104 (2018). https://doi.org/10.1126/sciadv.aau2104.

[3] X. Hong, Z. Xu, Z.-P. Lv, Z. Lin, M. Ahmadi, et al., High-permittivity solvents increase MXene stability and stacking order enabling ultraefficient terahertz shielding. Adv. Sci. **11**, 2305099 (2024). https://doi.org/10.1002/advs.202305099.

[4] J. Huang, S. Yang, X. Tang, L. Yang, W. Chen, et al., Flexible, transparent, and wafer-scale artificial synapse array based on $TiO_x/Ti_3C_2T_x$ film for neuromorphic computing. Adv. Mater. **35**, 2303737 (2023). https://doi.org/10.1002/adma.202303737.

[5] N. Wu, Y. Yang, C. Wang, Q. Wu, F. Pan, et al., Ultrathin cellulose nanofiber assisted ambient-pressure-dried, ultralight, mechanically robust, multifunctional MXene aerogels. Adv. Mater. **35**, 2207969 (2023). https://doi.org/10.1002/adma.202207969.

[6] J. Liu, H.-B. Zhang, R. Sun, Y. Liu, Z. Liu, et al., Hydrophobic, flexible, and lightweight MXene foams for high-performance electromagnetic-interference shielding. Adv. Mater. **29**, 1702367 (2017). https://doi.org/10.1002/adma.201702367.

[7] T. Zhou, Y. Yu, B. He, Z. Wang, T. Xiong, et al., Ultra-compact MXene fibers by continuous and controllable synergy of interfacial interactions and thermal drawing-

induced stresses. Nat. Commun. **13**, 4564 (2022). https://doi.org/10.1038/s41467-022-32361-6.

[8] X. Wu, T. Tu, Y. Dai, P. Tang, Y. Zhang, et al., Direct ink writing of highly conductive MXene frames for tunable electromagnetic interference shielding and electromagnetic wave-induced thermochromism. Nano-Micro Lett. **13**, 148 (2021). https://doi.org/10.1007/s40820-021-00665-9.

[9] S. Yang, R. Yang, Z. Lin, X. Wang, S. Liu, et al., Ultrathin, flexible, and high-strength polypyrrole/$Ti_3C_2T_x$ film for wide-band gigahertz and terahertz electromagnetic interference shielding. J. Mater. Chem. A **10**, 23570 (2022). https://doi.org/10.1039/D2TA06805B.

[10] S. Wan, Y. Chen, C. Huang, Z. Huang, C. Liang, et al., Scalable ultrastrong MXene films with superior osteogenesis. Nature **634**, 1103 (2024). https://doi.org/10.1038/s41586-024-08067-8.

[11] C. Pavlou, M. G. Pastore Carbone, A. C. Manikas, G. Trakakis, C. Koral, et al., Effective EMI shielding behaviour of thin graphene/PMMA nanolaminates in the THz range. Nat. Commun. **12**, 4655 (2021). https://doi.org/10.1038/s41467-021-24970-4.

[12] H. Wang, X. Sun, Y. Wang, K. Li, J. Wang, et al., Acid enhanced zipping effect to densify MWCNT packing for multifunctional MWCNT films with ultra-high electrical conductivity. Nat. Commun. **14**, 380 (2023). https://doi.org/10.1038/s41467-023-36082-2.

[13] S. Wan, Y. Chen, Y. Wang, G. Li, G. Wang, et al., Ultrastrong graphene films *via* long-chain π-bridging. Matter **1**, 389 (2019). https://doi.org/10.1016/j.matt.2019.04.006.

[14] D. Tao, C. Yang, C. Chen, K. Yan, H. You, et al., Highly flexible and ultralight PVA-co-PE-AgNW/MXene composite film with low filling for multistage electromagnetic interference shielding. Small **21**, 2411752 (2025). https://doi.org/10.1002/smll.202411752.

[15] G.-M. Weng, J. Li, M. Alhabeb, C. Karpovich, H. Wang, et al., Layer-by-layer assembly of cross-functional semi-transparent MXene-carbon nanotubes composite films for next-generation electromagnetic interference shielding. Adv. Funct. Mater. **28**, 1803360 (2018). https://doi.org/10.1002/adfm.201803360.

[16] F. Fang, Y.-Q. Li, H.-M. Xiao, N. Hu & S.-Y. Fu, Layer-structured silver nanowire/polyaniline composite film as a high performance X-band EMI shielding material. J. Mater. Chem. C **4**, 4193 (2016). https://doi.org/10.1039/C5TC04406E.
